# Vitamin D receptor gene polymorphisms and the risk of the type 1 diabetes: a meta-regression and updated meta-analysis

**DOI:** 10.1186/s12902-020-00575-8

**Published:** 2020-08-08

**Authors:** Na Zhai, Ramtin Bidares, Masoud Hassanzadeh Makoui, Saeed Aslani, Payam Mohammadi, Bahman Razi, Danyal Imani, Mohammad Yazdchi, Haleh Mikaeili

**Affiliations:** 1 Endocrine 1st Department, Baoding No.1 Central Hospital, Baoding, 071000 China; 2 Department of Experimental Medicine, Sapienza University, Rome, Italy; 3 Department of Immunology, School of Public Health, Tehran University of Medical Sciences, Tehran, Iran; 4 Department of Immunology, School of Medicine, Tehran University of Medical Sciences, Tehran, Iran; 5 Department of Clinical Biochemistry, School of Medicine, Tarbiat Modares University, Tehran, Iran; 6 Department of Hematology, School of Medicine, Tarbiat Modares University, Tehran, Iran; 7 Neuroscience Research Center, Tabriz University of Medical Sciences, Tabriz, Iran; 8 Tuberculosis and lung Diseases Research Center, Tabriz University of medical sciences, Tabriz, Iran

**Keywords:** Vitamin D receptor, Type 1 diabetes mellitus, Polymorphism, Meta-analysis

## Abstract

**Background:**

The association between the polymorphisms in the vitamin D receptor (*VDR*) gene and the risk of type 1 diabetes mellitus (T1DM) has been evaluated in several studies. However, the findings were inconclusive. Thus, we conducted a meta-analysis to comprehensively evaluate the effect of *VDR* gene polymorphisms on the risk of T1DM.

**Methods:**

All relevant studies reporting the association between *VDR* gene polymorphisms and susceptibility to T1DM published up to May 2020 were identified by comprehensive systematic database search in ISI Web of Science, Scopus, and PubMed/MEDLINE. Strength of association were assessed by calculating of pooled odds ratios (ORs) and 95% confidence intervals (CIs). The methodological quality of each study was assessed according to the Newcastle–Ottawa Scale. To find the potential sources of heterogeneity, meta-regression and subgroup analysis were also performed.

**Results:**

A total of 39 case–control studies were included in this meta-analysis. The results of overall population rejected any significant association between *VDR* gene polymorphisms and T1DM risk. However, the pooled results of subgroup analysis revealed significant negative and positive associations between *FokI* and *BsmI* polymorphisms and T1DM in Africans and Americans, respectively.

**Conclusions:**

This meta-analysis suggested a significant association between *VDR* gene polymorphism and T1DM susceptibility in ethnic-specific analysis.

## Background

Type 1 diabetes mellitus (T1DM) is a globally-widespread disease that is characterized by a reduction in insulin production or the production of ineffective insulin [1]. It is generally believed that the immune-associated destruction of beta cells of the islets of Langerhans causes the disease, resulting in lower insulin levels (that is called type 1a diabetes mellitus). In a smaller T1DM subset, no evidence of autoimmunity can be found (type 1b) [2]. T1DM constitutes roughly 5 to 10% of all diabetes cases, and its prevalence is still rising [3]. With more than half a million children living with T1DM, and almost 90,000 children diagnosed each year, T1DM inflicts mostly children of under 15 years of age [4]. It is well known that T1DM is a multi-factorial autoimmune disorder caused by interactions between genetic and environmental factors [5].

Vitamin D (VitD) is a steroid molecule that has many roles in the body, such as regulation of the immune cells. In addition to immune responses, VitD is also involved in the etiopathogenesis of several disorders, such as cancer, autoimmune disorders, cardiovascular disorders, asthma, and diabetes [6–9]. In animal model of T1DM, VitD suppresses the occurrence of diabetes, by regulating the T helper (Th) 1/Th2 cytokine balance in the local pancreatic lesions [10, 11]. Moreover, VitD inhibits T cell activation and secretion of pro-inflammatory cytokines, such as interleukin (IL)-1, IL-6, IL-12, tumor necrosis factor (TNF)-α, and interferon (IFN)-γ, which are involved in the pathogenesis of T1DM [12–14]. Mostly, VitD exerts its function through vitamin D receptor (VDR), which is found in the nuclei of target cells, such as lymphocytes, macrophages, and pancreatic cells. VDR is a member of the nuclear hormone receptors superfamily and has been linked to insulin sensitivity and secretion [15].

Four common single nucleotide polymorphisms (SNPs) of VDR gene are FokI (rs2228570), TaqI (rs731236), BsmI (rs1544410), and ApaI (rs7975232). Among them, ApaI, BsmI, and TaqI polymorphisms are located in the 3′-end of *VDR* gene which lead to silent mutation associated with increased *VDR* mRNA stability. In contrast, FokI SNP is located in the start codon that produces a protein with shorter size (424 amino acids), which is more active than the long form (427 amino acids) [8, 16, 17]. Over the course of past few decades, the VDR gene polymorphisms have been associated with susceptibility to numerous autoimmune disorders [8, 18, 19].

In recent years, several studies have investigated the association between *VDR* gene SNPs and T1DM in all over the world, which have yielded conflicting results. The reasons for these discrepancies might be small sample sizes, clinical heterogeneity, and low statistical power. Therefore, a comprehensive meta-analysis might be the best way to solve these problems. Two previous meta-analyses performed by Tizaouia et al. in 2014 [20] and Guo et al. in 2006 [21] reported that *VDR* gene polymorphisms were not associated with the susceptibility to T1DM. However, Zhang et al. in 2012 [22] demonstrated that BsmI polymorphism was significantly associated with the risk of T1DM. Furthermore, Sahin et al. in 2017 indicated that BsmI and TaqI polymorphisms were associated withT1DM risk in children with less than average 15 years old [23]. Qin et al. in 2014 evaluated the association of only BsmI SNP with T1DM risk and demonstrated its association in the overall analysis, as well as in Asians, Latino, and Africans [24]. In 2014, Wang et al., by including 20 studies, reported that BsmI polymorphism might be a risk factor for susceptibility to T1DM in the East Asian population, and the FokI polymorphism was associated with an increased risk of T1DM in the West Asian population [25].

Since several articles published after the last meta-analysis, here we conducted an updated meta-analysis with the aim of providing a much more reliable conclusion on the significance of the association between *VDR* gene polymorphisms and T1DM risk.

## Methods

This meta-analysis was conducted according to the Preferred Reporting Items for Systematic reviews and Meta-Analyses (PRISMA) guidelines, including search strategy, inclusion and exclusion criteria, data extraction and quality assessment, and statistical analysis [26].

### Search strategy

Three electronic databases (PubMed/MEDLINE, Scopus, and Web of Science) were systematically searched for studies regarding the association of *VDR* gene polymorphisms, including FokI (rs2228570) and/or TaqI (rs731236) and/or BsmI (rs1544410) and/or ApaI (rs7975232), and T1DM susceptibility, which were published before May 2020. The following combinations of search terms were used: (“T1D” OR “type 1 diabetes” OR “diabetes”) AND (“VDR” OR “vitamin D receptor”) AND (“polymorphisms” OR “SNP” OR “variation” OR “mutation”). The reference lists of review articles were also manually searched for additional pertinent publications. Original data in English language and human population studies were collected.

### Inclusion and exclusion criteria

Eligible studies must meet the following criteria: a) All studies assessing the association of *VDR* gene polymorphisms and T1DM risk; b) All studies reporting sufficient data to calculate the odds ratio (OR) and its 95% confidence intervals (CIs); c) All studies with distinct case and control groups (case-control and cohort design). The exclusion criteria were: a) studies that their genotype or allele frequency could not be extracted; b) letters, non-English publications, animal studies, case reports, reviews, comments, book chapters, and abstracts; c) duplicate and republished studies. The application of these criteria recognized 39 studies eligible for the quantitative analysis.

### Data extraction and quality assessment

According to a standardized extraction form, the following data were independently extracted by two reviewers: the author’s name, journal and year of publication, country of origin, ethnicity, number of case and control for each gender separately, genotype and allele frequencies in cases and healthy groups, mean or range of age, genotyping method, total sample size of cases and controls. The third reviewer finalized the extracted data, and potential discrepancies were resolved by consensus. For quality assessment of the included publications, the Newcastle-Ottawa Scale (NOS) was applied [27]. In this respect, studies with 0–3, 4–6 or 7–9 scores were, respectively, of low, moderate, and high-quality.

### Statistical analysis

Deviation from Hardy–Weinberg equilibrium (HWE) for distribution of the allele frequencies was analyzed by χ2-test in control groups. The strength of association between *VDR* gene polymorphisms and T1DM susceptibility was estimated by calculating pooled OR and its 95% CI. Different comparison model for FokI, TaqI, BsmI, and ApaI were as follows: ***FokI***; dominant model (ff + Ff vs. FF), recessive model (ff vs. Ff + FF), allelic model (f vs. F), homozygote (ff vs. FF), and heterozygote (Ff vs. FF): ***TaqI***; dominant model (tt + Tt vs. TT), recessive model (tt vs. Tt + TT), allelic model (t vs. T), homozygote (tt vs. TT), and heterozygote (Tt vs. TT): ***BsmI***; dominant model (bb + Bb vs. BB), recessive model (bb vs. Bb + BB), allelic model (b vs. B), homozygote (bb vs. BB), and heterozygote (Bb vs. BB): ***ApaI***; dominant model (aa+Aa vs. AA), recessive model (aa vs. Aa+AA), allelic model (a vs. A), homozygote (aa vs. AA), and heterozygote (Aa vs. AA). The heterogeneity among studies was measured by the χ2 test-based Q statistic, and I^2^ value which quantify the degree of heterogeneity [28]. Accordingly, heterogeneity was considered significant if I^2^ values exceeded 50% or the Q statistic had a *P* value of less than 0.1 and random-effects model (DerSimonian–Laird approach) was carried out [29]. Otherwise, the fixed-effects model (Mantel–Haenszel approach) was performed for combination of data [30]. In order to assess the predefined sources of heterogeneity among included studies, subgroup analysis and meta-regression analysis based on year of population, and ethnicity were performed. Stability of our results was assessed by sensitivity analysis. Potential publication bias was estimated by Egger’s linear regression test, and also Begg’s test was employed to estimate the funnel plot asymmetry (*P* value< 0.05 considered statistically significant) [31, 32]. The data analyses were carried out using STATA (version 14.0; Stata Corporation, College Station, TX) and SPSS (version 23.0; SPSS, Inc. Chicago, IL).

## Results

### Study characteristics

**Fig. 1:**
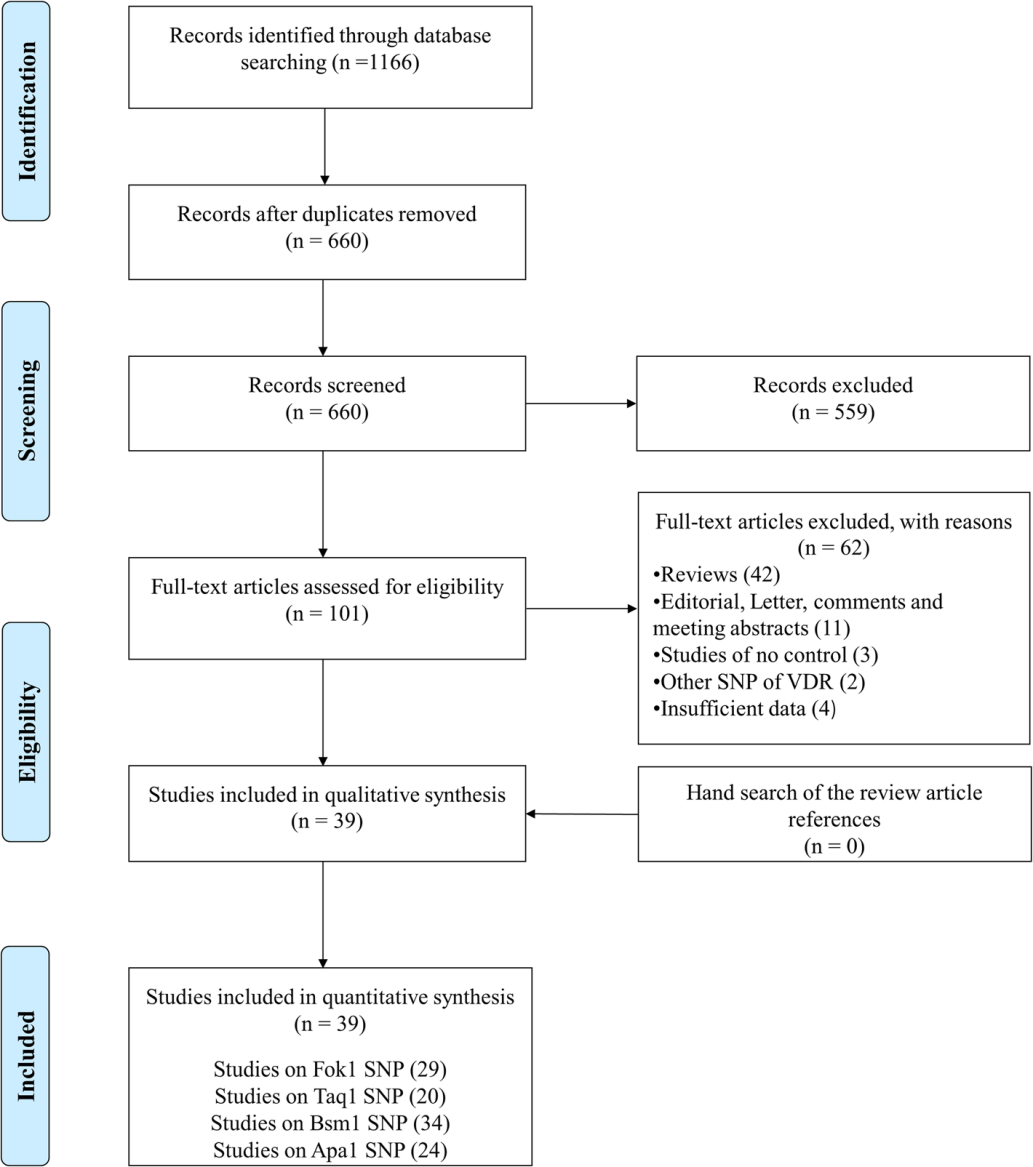
Flow diagram of study selection process

Regarding to aforementioned keywords, a total of 1116 studies were initially retrieved. Of these studies, 456 publications were duplicate, 559 and 62 publications excluded by title & abstract and full text examination, respectively. Finally, 39 studies qualified for quantitative analysis. It should be noted that while the latest meta-analysis by Tizaouia et al. [20] in 2014 included 23 studies, we performed the updated meta-analysis by adding 16 more articles. Also, no studies were found by hand search (Fig. 1**)**. The eligible studies were published from 1998 to 2019 and had an overall good methodological quality with NOS scores ranging from 6 to 8. Polymerase chain reaction-restriction fragment length polymorphism (PCR- RFLP) and Taq-man were used by majority of included studies as genotyping method. Tables 1 and 2 summarized the characteristics and genotype frequency of the included studies.

**Table 1:** Characteristics of studies included in meta-analysis of overall T1DM

Study author	Year	Country	Ethnicity	Sex cases/controls	Total cases/control	Age case/control (Mean)	Genotyping method	Quality score
**FokI (rs2228570)**								
Ban et al. [33]	2001	Japan	Asian	M = 50/60 F = 100/150	108 / 250	26.0 ± 3.8 / NR	RFLP-PCR	7
Fassbender et al. [34]	2002	Germany	European	M = 42/33 F = 27/30	75 / 57	34.1 ± 11.1 / 33.5 ± 10.1	RFLP-PCR	6
Gyorffy et al. [35]	2002	Hungary	European	M = 57/50 F = 53/50	107 / 103	23.5 ± 5.11 / NR	RFLP-PCR	7
Turpeinen (Turku) et al. [36]	2003	Finland	European	M = NR F=NR	274 / 808	NR / NR	Mini sequencing	8
Turpeinen (Tampere) et al. [36]	2003	Finland	European	M = NR F=NR	55 / 457	NR / NR	Mini sequencing	8
Turpeinen (Oulu) et al. [36]	2003	Finland	European	M = NR F=NR	249 / 795	NR / NR	Mini sequencing	8
Audi (barcellona) et al. [37]	2004	Spain	European	M = 69/86 F = 153/122	155 / 275	NR / NR	Mini sequencing	7
Audi (navarra) et al. [37]	2004	Spain	European	M = 40/46 F = 58/58	86 / 116	NR / NR	Mini sequencing	7
San Pedro et al. [38]	2005	Spain	European	M = NR F=NR	71 / 88	14.5 ± 9.9 / NR	RFLP-PCR	6
Zemunik et al. [39]	2005	Croatia	European	M = 72/62 F=NR	134 / 232	8.6 ± 4.3 / NR	RFLP-PCR	7
Capoluongo et al. [40]	2006	Italy	European	M = 135/111 F = 135/111	246 / 246	39.3 ± 11.1 / 39.6 ± 9.1	RFLP-PCR	8
Lemos et al. [41]	2008	Portugal	European	M = 113/94 F = 143/106	207 / 249	27.5 ± 10.2 / 36.8 ± 13.8	RFLP-PCR	8
Israni et al. [42]	2009	India	Asian	M = 131/135 F = 116/81	236 / 197	15.1 ± 7.30 / 30.1 ± 10.2	RFLP-PCR	7
Mory et al. [43]	2009	Brazil	American	M = NR F=NR	177 / 182	17.2 ± 5.4 / 12.2 ± 8.1	RFLP-PCR	7
Panierakis et al. [15]	2009	Greece	European	M = NR F = 52/44	100 / 96	NR / NR	Mini sequencing	6
Yavuz et al. [44]	2011	turkey	European	M = 60/57 F = 73/61	117 / 134	27.6 ± 7.3 / 26.2 ± 5.3	RFLP-PCR	6
Yokota et al. [45]	2012	Japan	Asian	M = NR F=NR	108 / 220	NR / NR	NR	7
Bonakdaran et al. [46]	2012	Iran	Asian	M = 28/41 F = 19/26	69 / 45	NR / NR	RFLP-PCR	6
Sahin et al. [47]	2012	Turkey	European	M = NR F=NR	85 / 80	NR / NR	NR	6
Mohammadnejad et al. [48]	2012	Iran	Asian	M = 32/55 F = 50/50	87 / 100	27.93 ± 10.86 / 28.58 ± 7.40	RFLP-PCR	6
Vedralova et al. [49]	2012	Czech	European	M = NR F=NR	116 / 113	67.0 ± 12.44 / 45.0 ± 7.31	RFLP-PCR	6
Greer et al. [50]	2012	Australia	Australian	M = NR F=NR	50 / 55	NR / NR	RFLP-PCR	6
Hamed et al. [51]	2013	Egypt	African	M = 64/68 F = 18/22	132 / 40	8.5 ± 3.3 / 9.0 ± 1.5	RFLP-PCR	6
Abd-Allah et al. [52]	2014	Egypt	African	M = 42/78 F = 42/78	120 / 120	11.7 ± 2.8 / 11.1 ± 2.6	RFLP-PCR	7
Kafoury et al. [53]	2014	Egypt	African	M = 25/35 F=NR	60 / 60	11.2 ± 3.7 / 27.2 ± 6.4	RFLP-PCR	6
Nasreen et al. [54]	2016	Pakistan	Asian	M = 25/19 F = 23/21	44 / 44	14.81 ± 2.7 / 17.92 ± 2.8	RFLP-PCR	6
Mukhtar et al. [55]	2017	Pakistan	Asian	M = NR F=NR	102 / 100	13/2 / 13/8	RFLP-PCR	6
Ali et al. [56]	2018	Saudi Arabia	Asian	M = 54/46 F = 43/59	100 / 102	10.33 ± 3.15 / > 35	RFLP-PCR	7
Rasoul et al. [57]	2019	Kuwait	Asian	M = NR F=NR	253 / 214	8.5 ± 5.5 / 8.9 ± 5.2	RFLP-PCR	8
**TaqI (rs731236)**								
Chang et al. [58]	2000	China	Asian	M = 71/86 F = 156/92	157 /248	23.5 ± 5.11 / 32.4 ± 6.6	RFLP-PCR	8
Fassbender et al. [34]	2002	Germany	European	M = 57/50 F = 53/50	75 /57	5.8 ± 2.3 / NR	RFLP-PCR	6
Gyorffy et al. [35]	2002	Hungary,	European	M = 57/50 F = 53/50	107 / 103	23.5 ± 5.11 / NR	RFLP-PCR	7
Skrabic et al. [59]	2003	Croatia	European	M = 72/62 F = 60/72	134 / 132	8.69 ± 4.3 / 8.24 ± 4.9	RFLP-PCR	7
Bianco et al. [60]	2004	Italy	European	M = NR F=NR	31 / 36	NR / NR	RFLP-PCR	6
San Pedro et al. [38]	2005	Spain	European	M = NR F=NR	71 / 88	14.5 ± 9.9 / NR	RFLP-PCR	6
Garcia et al. [61]	2007	Chile	American	M = 120/96 F = 106/97	216 / 203	9.3 ± 4.2 / 10.3 ± 2.5	RFLP-PCR	8
Lemos et al. [41]	2008	Portugal	European	M = NR F=NR	205 / 232	27.5 ± 10.2 / 36.8 ± 13.8	RFLP-PCR	8
Israni et al. [42]	2009	India	Asian	M = 131/135 F = 116/81	236 / 197	15.1 ± 7.30 / 30.1 ± 10.2	RFLP-PCR	7
Panierakis et al. [15]	2009	Greece	European	M = NR F = 52/44	100 / 96	NR / NR	Mini sequencing	6
Yavuz et al. [44]	2011	Turkey	European	M = 60/57 F = 73/61	117 / 134	27.6 ± 7.3 / 26.2 ± 5.3	RFLP-PCR	6
Bonakdaran et al. [46]	2012	Iran	Asian	M = 28/41 F = 19/26	69 / 45	NR / NR	RFLP-PCR	6
Mohammadnejad et al. [48]	2012	Iran	Asian	M = 32/55 F = 50/50	87 / 100	27.93 ± 10.86 / 28.58 ± 7.40	RFLP-PCR	6
Greer et al. [50]	2012	Australia	Australian	M = NR F=NR	50 / 55	NR / NR	RFLP-PCR	6
Abd-Allah et al. [52]	2014	Egypt	African	M = 42/78 F = 42/78	120 / 120	11.7 ± 2.8 / 11.1 ± 2.6	RFLP-PCR	7
Cheon et al. [62]	2015	Korea	Asian	M = 35/46 F = 53/60	81 / 113	10.28 ± 3.73 / 9.98 ± 3.56	RFLP-PCR	6
Khalid et al. [63]	2016	Saudi Arabia	Asian	M = NR F=NR	100 / 50	11.48 ± 3.39 / 9.50 ± 4.23	RFLP-PCR	6
Iyer et al. [64]	2017	Saudi Arabia	Asian	M = 25/25 F = 25/25	50 / 50	25.37 ± 4.07 / 23.44 ± 5.38	RFLP-PCR	6
Rasoul et al. [57]	2019	Kuwait	Asian	M = NR F=NR	253 / 214	8.5 ± 5.5 / 8.9 ± 5.2	RFLP-PCR	8
Ahmed et al. [65]	2019	Egypt	African	M = 24/25 F = 26/25	50 / 50	11.16 ± 3.27 / 10.97 ± 2.77	RFLP-PCR	6
**BsmI (rs1544410)**								
Hauache et al. [66]	1998	Brazil	American	M = NR F = 31/63	78 / 94	15.5 ± 6.0 / 49 ± 11	RFLP-PCR	6
Chang et al. [58]	2000	China	Asian	M = 71/86 F = 156/92	157 / 248	23.5 ± 5.11 / 32.4 ± 6.6	RFLP-PCR	8
Fassbender et al. [34]	2002	Germany	European	M = 57/50 F = 53/50	75 / 57	5.8 ± 2.3 / NR	RFLP-PCR	6
Gyorffy et al. [35]	2002	Hungary	European	M = 57/50 F = 53/50	107 / 103	23.5 ± 5.11 / NR	RFLP-PCR	7
Motohashi et al. [67]	2002	Japan	Asian	M = 96/107 F = 101/121	203 / 222	34.6 ± 16.9 / 44.4 ± 13.7	RFLP-PCR	8
Skrabic et al. [59]	2003	Croatia	European	M = 72/62 F = 60/72	134 / 132	8.69 ± 4.3 / 8.24 ± 4.9	RFLP-PCR	7
Turpeinen (Turku) et al. [36]	2003	Finland	European	M = NR F=NR	220 / 844	NR / NR	Mini sequencing	8
Turpeinen (Tampere) et al. [36]	2003	Finland	European	M = NR F=NR	58 / 1175	NR / NR	Mini sequencing	8
Turpeinen (Oulu) et al. [36]	2003	Finland	European	M = NR F=NR	226 / 818	NR / NR	Mini sequencing	8
Audi (barcellona) et al. [37]	2004	Spain	European	M = 69/84 F = 153/121	153 / 274	NR / NR	Mini sequencing	7
Audi (navarra) et al. [37]	2004	Spain	European	M = 40/49 F = 58/58	89 /116	NR / NR	Mini sequencing	7
Bianco et al. [60]	2004	Italy	European	M = NR F=NR	31 / 36	NR / NR	RFLP-PCR	6
San Pedro et al. [38]	2005	Spain	European	M = NR F=NR	71 / 88	14.5 ± 9.9 / NR	RFLP-PCR	6
Capoluongo et al. [40]	2006	Italy	European	M = 135/111 F = 135/111	246 / 246	39.3 ± 11.1 / 39.6 ± 9.1	RFLP-PCR	8
Garcia et al. [61]	2007	Chile	American	M = NR F = 106/97	208 / 203	9.3 ± 4.2 / 10.3 ± 2.5	RFLP-PCR	8
Lemos et al. [41]	2008	Portugal	European	M = NR F=NR	207 / 248	27.5 ± 10.2 / 36.8 ± 13.8	RFLP-PCR	8
Shimada et al. [68]	2008	Japan	Asian	M = 357/417 F=NR	774 / 599	29/8 / NR	RFLP-PCR	8
Israni et al. [42]	2009	India	Asian	M = 131/135 F = 116/81	236 / 197	15.1 ± 7.30 / 30.1 ± 10.2	RFLP-PCR	7
Mory et al. [43]	2009	Brazil	American	M = NR F=NR	177 / 182	17.2 ± 5.4 / 12.2 ± 8.1	RFLP-PCR	7
Panierakis et al. [15]	2009	Greece	European	M = NR F = 52/44	100 / 96	NR / NR	Mini sequencing	6
Yavuz et al. [44]	2011	Turkey	European	M = 60/57 F = 73/61	117 / 134	27.6 ± 7.3 / 26.2 ± 5.3	RFLP-PCR	6
Tawfeek et al. [69]	2011	Arabic Saudi	Asian	M = 0/30 F = 0/14	30 / 14	35.7 ± 5.33 / 33.2 ± 4.06	RFLP-PCR	6
Bonakdaran et al. [46]	2012	Iran	Asian	M = 28/41 F = 19/26	69 / 45	NR / NR	RFLP-PCR	6
Vedralova et al. [49]	2012	Czech	European	M = NR F=NR	104 / 83	67.0 ± 12.44 / 45.0 ± 7.31	RFLP-PCR	6
Mohammadnejad et al. [48]	2012	Iran	Asian	M = 32/55 F = 50/50	87 / 100	27.93 ± 10.86 / 28.58 ± 7.40	RFLP-PCR	6
Moubarak et al. [70]	2013	Syria	Asian	M = 25/30 F = 24/26	55 / 50	13.75 ± 6.91 / 39.86 ± 11.66	RFLP-PCR	6
Abd-Allah et al. [52]	2014	Egypt	Africian	M = 42/78 F = 42/78	120 / 120	11.7 ± 2.8 / 11.1 ± 2.6	RFLP-PCR	7
Kafoury et al. [53]	2014	Egypt	Africian	M = 25/35 F=NR	60 / 56	11.2 ± 3.7 / 27.2 ± 6.4	RFLP-PCR	6
Cheon et al. [62]	2015	Korea	Asian	M = 35/46 F = 53/60	81 / 113	10.28 ± 3.73 / 9.98 ± 3.56	RFLP-PCR	6
Khalid et al. [63]	2016	Saudi Arabia	Asian	M = NR F=NR	100 / 50	11.48 ± 3.39 / 9.50 ± 4.23	RFLP-PCR	6
Iyer et al. [64]	2017	Saudi Arabia	Asian	M = 25/25 F = 25/25	50 / 50	25.37 ± 4.07 / 23.44 ± 5.38	RFLP-PCR	6
Ali et al. [56]	2018	Saudi Arabia	Asian	M = 54/46 F = 43/59	100 / 102	10.33 ± 3.15 / > 35	RFLP-PCR	7
Rasoul et al. [57]	2019	Kuwait	Asian	M = NR F=NR	253 / 214	8.5 ± 5.5 / 8.9 ± 5.2	RFLP-PCR	8
Ahmed et al. [65]	2019	Egypt	African	M = 24/25 F = 26/25	50 / 50	11.16 ± 3.27 / 10.97 ± 2.77	RFLP-PCR	6
**ApaI (rs7975232)**								
Chang et al. [58]	2000	China	Asian	M = 71/86 F = 156/92	157 / 248	23.5 ± 5.11 / 32.4 ± 6.6	RFLP-PCR	8
Gyorffy et al. [35]	2002	Hungary	European	M = 57/50 F = 53/50	107 / 103	23.5 ± 5.11 / NR	RFLP-PCR	7
Skrabic et al. [59]	2003	Croatia	European	M = 72/62 F = 60/72	134 / 132	8.69 ± 4.3 / 8.24 ± 4.9	RFLP-PCR	7
Turpeinen (Turku) et al. [36]	2003	Finland	European	M = NR F=NR	198 / 797	NR / NR	Mini sequencing	8
Turpeinen (Tampere) et al. [36]	2003	Finland	European	M = NR F=NR	56 / 450	NR / NR	Mini sequencing	8
Turpeinen (Oulu) et al. [36]	2003	Finland	European	M = NR F=NR	239 / 843	NR / NR	Mini sequencing	8
Bianco et al. [60]	2004	Italy	European	M = NR F=NR	31 / 36	NR / NR	RFLP-PCR	6
San Pedro et al. [38]	2005	Spain	European	M = NR F=NR	71 / 88	14.5 ± 9.9 / NR	RFLP-PCR	6
Garcia et al. [61]	2007	Chile	American	M = NR F = 106/97	213 / 203	9.3 ± 4.2 / 10.3 ± 2.5	RFLP-PCR	8
Lemos et al. [41]	2008	Portugal	European	M = NR F=NR	205 / 232	27.5 ± 10.2 / 36.8 ± 13.8	RFLP-PCR	8
Israni et al. [42]	2009	India	Asian	M = 131/135 F = 116/81	236 / 197	15.1 ± 7.30 / 30.1 ± 10.2	RFLP-PCR	7
Panierakis et al. [15]	2009	Greece	European	M = NR F = 52/44	100 / 96	NR / NR	Mini sequencing	6
Yavuz et al. [44]	2011	Turkey	European	M = 60/57 F = 73/61	117 / 136	27.6 ± 7.3 / 26.2 ± 5.3	RFLP-PCR	6
Bonakdaran et al. [46]	2012	Iran	Asian	M = 28/41 F = 19/26	69 / 45	NR / NR	RFLP-PCR	6
Mohammadnejad et al. [48]	2012	Iran	Asian	M = 32/55 F = 50/50	87 / 100	27.93 ± 10.86 / 28.58 ± 7.40	RFLP-PCR	6
Greer et al. [50]	2012	Australia	Australian	M = NR F=NR	50 / 55	NR / NR	RFLP-PCR	6
Abd-Allah et al. [52]	2014	Egypt	African	M = 42/78 F = 42/78	120 / 120	11.7 ± 2.8 / 11.1 ± 2.6	RFLP-PCR	7
Cheon et al. [62]	2015	Korea	Asian	M = 35/46 F = 53/60	81 / 113	10.28 ± 3.73 / 9.98 ± 3.56	RFLP-PCR	6
Khalid et al. [63]	2016	Saudi Arabia	Asian	M = NR F=NR	100 / 50	11.48 ± 3.39 / 9.50 ± 4.23	RFLP-PCR	6
Nasreen et al. [54]	2016	Pakistan	Asian	M = 25/19 F = 23/21	44 / 44	14.81 ± 2.7 / 17.92 ± 2.8	RFLP-PCR	6
Iyer et al. [64]	2017	Saudi Arabia	Asian	M = 25/25 F = 25/25	50 / 50	25.37 ± 4.07 / 23.44 ± 5.38	RFLP-PCR	6
Mukhtar et al. [55]	2017	Pakistan	Asian	M = NR F=NR	102 / 100	13/2 / 13/8	RFLP-PCR	6
Rasoul et al. [57]	2019	Kuwait	Asian	M = NR F=NR	252 / 214	8.5 ± 5.5 / 8.9 ± 5.2	RFLP-PCR	8
Ahmed et al. [65]	2019	Egypt	African	M = 24/25 F = 26/25	50 / 50	11.16 ± 3.27 / 10.97 ± 2.77	RFLP-PCR	6

*NR* not reported, *M* male, *F* female

**Table 2:** Distribution of genotype and allele among T1DM patients and controls

Study author	T1DM cases					Healthy control					P-HWE	MAF
	FF	Ff	ff	F	f	FF	Ff	Ff	F	f		
**FokI (rs2228570)**												
Ban et al. [33]	50	52	6	152	64	82	138	30	302	198	0.01	0.396
Fassbender et al. [34]	35	30	10	100	50	19	30	8	68	46	0.48	0.403
Gyorffy et al. [35]	32	56	19	120	94	34	47	22	115	91	0.44	0.441
Turpeinen (Turku) et al. [36]	50	150	74	250	298	102	414	292	618	998	0.01	0.617
Turpeinen (Tampere) et al. [36]	7	28	20	42	68	61	226	170	348	566	0.29	0.619
Turpeinen (Oulu) et al. [36]	37	114	98	188	310	93	360	342	546	1044	0.9	0.656
Audi (barcellona) et al. [37]	69	68	18	206	104	105	142	28	352	198	0.04	0.36
Audi (navarra) et al. [37]	35	45	6	115	57	41	53	22	135	97	0.51	0.418
San Pedro et al. [38]	31	35	5	97	45	41	39	8	121	55	0.76	0.312
Zemunik et al. [39]	42	63	29	147	121	73	136	23	282	182	< 0.001	0.392
Capoluongo et al. [40]	89	112	45	290	202	91	127	28	309	183	0.09	0.371
Lemos et al. [41]	81	101	25	263	151	97	114	38	308	190	0.63	0.381
Israni et al. [42]	142	79	15	363	109	116	76	5	308	86	0.06	0.218
Mory et al. [43]	80	81	16	241	113	91	67	24	249	115	0.04	0.315
Panierakis et al. [15]	50	43	7	143	57	64	31	1	159	33	0.18	0.171
Yavuz et al. [44]	61	46	10	168	66	60	63	11	183	85	0.32	0.317
Yokota et al. [45]	50	46	12	146	70	59	20	141	138	302	< 0.001	0.686
Bonakdaran et al. [46]	38	25	6	101	37	18	20	7	56	34	0.71	0.377
Sahin et al. [47]	54	31	0	139	31	43	28	9	114	46	0.19	0.287
Mohammadnejad et al. [48]	49	33	5	131	43	55	40	5	150	50	0.5	0.25
Vedralova et al. [49]	38	60	18	136	96	25	76	12	126	100	< 0.001	0.442
Greer et al. [50]	21	21	8	63	37	28	22	5	78	32	0.82	0.29
Hamed et al. [51]	24	92	16	140	124	8	28	4	44	36	0.008	0.45
Abd-Allah et al. [52]	58	50	12	166	74	78	38	4	194	46	0.8	0.191
Kafoury et al. [53]	23	21	16	67	53	41	12	7	94	26	0.001	0.216
Nasreen et al. [54]	32	12	0	76	12	25	19	0	69	19	0.06	0.215
Mukhtar et al. [55]	84	13	5	181	23	100	0	0	200	0	< 0.001	0
Ali et al. [56]	64	33	3	161	39	79	21	2	179	25	0.66	0.122
Rasoul et al. [57]	178	30	45	386	120	146	67	1	359	69	0.02	0.161
**Study author**	**T1DM cases**					**Healthy control**					**P-HWE**	**MAF**
	**TT**	**Tt**	**tt**	**T**	**t**	**TT**	**Tt**	**tt**	**T**	**t**		
**TaqI (rs731236)**												
Chang et al. [58]	142	15	0	299	15	233	14	1	480	16	0.13	0.032
Fassbender et al. [34]	34	31	10	99	51	19	20	18	58	56	0.02	0.491
Gyorffy et al. [35]	46	34	27	126	88	42	27	34	111	95	< 0.001	0.461
Skrabic et al. [59]	54	55	25	163	105	48	72	12	168	96	0.04	0.363
Bianco et al. [60]	10	18	3	38	24	11	20	5	42	30	0.39	0.416
San Pedro et al. [38]	24	36	11	84	58	31	43	14	105	71	0.88	0.403
Garcia et al. [61]	115	79	22	309	123	121	69	13	311	95	0.46	0.233
Lemos et al. [41]	70	94	41	234	176	91	95	46	277	187	0.02	0.403
Israni et al. [42]	91	112	33	294	178	80	98	19	258	136	0.15	0.345
Panierakis et al. [15]	34	59	7	127	73	10	64	22	84	108	< 0.001	0.562
Yavuz et al. [44]	37	58	22	132	102	41	66	27	148	120	0.96	0.447
Bonakdaran et al. [46]	34	28	7	96	42	20	17	8	57	33	0.21	0.366
Mohammadnejad et al. [48]	32	52	3	116	58	59	41	0	159	41	< 0.001	0.205
Greer et al. [50]	18	26	6	62	38	26	24	5	76	34	0.87	0.309
Abd-Allah et al. [52]	42	66	12	150	90	33	69	18	135	105	0.06	0.437
Cheon et al. [62]	66	15	0	147	15	105	8	0	218	8	0.69	0.035
Khalid et al. [63]	63	22	15	148	52	19	16	15	54	46	0.01	0.46
Iyer et al. [64]	19	14	17	52	48	16	16	18	48	52	0.01	0.52
Rasoul et al. [57]	96	96	61	288	218	156	36	22	348	80	< 0.001	0.186
Ahmed et al. [65]	0	42	8	42	58	0	40	10	40	60	< 0.001	0.6
**Study author**	**T1DM cases**					**Healthy control**					**P-HWE**	**MAF**
	**BB**	**Bb**	**bb**	**B**	**b**	**BB**	**Bb**	**bb**	**B**	**b**		
**BsmI (rs1544410)**												
Hauache et al. [66]	13	39	26	65	91	12	43	39	67	121	0.97	0.643
Chang et al. [58]	4	16	137	24	290	1	16	231	18	478	0.22	0.963
Fassbender et al. [34]	14	35	26	63	87	18	25	14	61	53	0.37	0.464
Gyorffy et al. [35]	19	46	42	84	130	16	53	34	85	121	0.53	0.587
Motohashi et al. [67]	12	64	127	88	318	1	49	172	51	393	0.2	0.885
Skrabic et al. [59]	24	58	52	106	162	17	74	41	108	156	0.06	0.59
Turpeinen (Turku) et al. [36]	97	97	26	291	149	354	388	102	1096	592	0.78	0.35
Turpeinen (Tampere) et al. [36]	29	22	7	80	36	533	488	154	1554	796	0.01	0.338
Turpeinen (Oulu) et al. [36]	90	103	33	283	169	403	305	110	1111	525	< 0.001	0.32
Audi (barcellona) et al. [37]	21	73	59	115	191	46	147	81	239	309	0.13	0.563
Audi (navarra) et al. [37]	20	43	26	83	95	19	53	44	91	141	0.65	0.607
Bianco et al. [60]	13	14	4	40	22	14	17	5	45	27	0.96	0.375
San Pedro et al. [38]	15	40	16	70	72	17	44	27	78	98	0.9	0.556
Capoluongo et al. [40]	62	125	59	249	243	61	122	63	244	248	0.89	0.504
Garcia et al. [61]	21	110	77	152	264	14	74	115	102	304	0.65	0.748
Lemos et al. [41]	43	96	68	182	232	56	107	85	219	277	0.04	0.558
Shimada et al. [68]	32	165	577	229	1319	7	121	471	135	1063	0.8	0.887
Israni et al. [42]	79	120	37	278	194	56	94	47	206	188	0.53	0.477
Mory et al. [43]	60	57	60	177	177	38	74	70	150	214	0.62	0.587
Panierakis et al. [15]	23	57	20	103	97	38	43	15	119	73	0.62	0.38
Yavuz et al. [44]	20	57	40	97	137	14	59	61	87	181	0.96	0.675
Tawfeek et al. [69]	3	18	9	24	36	1	8	5	10	18	0.36	0.642
Bonakdaran et al. [46]	14	26	29	54	84	16	11	18	43	47	< 0.001	0.522
Vedralova et al. [49]	43	47	14	133	75	30	33	20	93	73	0.07	0.439
Mohammadnejad et al. [48]	11	36	40	58	116	9	45	46	63	137	0.66	0.685
Moubarak et al. [70]	7	25	23	39	71	14	26	10	54	46	0.74	0.46
Abd-Allah et al. [52]	27	68	25	122	118	48	52	20	148	92	0.36	0.383
Kafoury et al. [53]	8	13	39	29	91	4	11	41	19	93	0.02	0.83
Cheon et al. [62]	0	13	68	13	149	1	4	108	6	220	< 0.001	0.973
Khalid et al. [63]	51	32	17	134	66	19	21	10	59	41	0.35	0.41
Iyer et al. [64]	8	12	30	28	72	26	12	12	64	36	< 0.001	0.36
Ali et al. [56]	30	45	25	105	95	62	28	12	152	52	0.005	0.254
Rasoul et al. [57]	141	83	29	365	141	120	66	28	306	122	< 0.001	0.285
Ahmed et al. [65]	8	35	7	51	49	32	18	0	82	18	< 0.001	0.19
**Study author**	**T1DM cases**					**Healthy control**					**P-HWE**	**MAF**
	**AA**	**Aa**	**aa**	**A**	**a**	**AA**	**Aa**	**aa**	**A**	**a**		
**ApaI (rs7975232)**												
Chang et al. [58]	16	76	65	108	206	13	105	130	131	365	0.16	0.735
Gyorffy et al. [35]	23	27	57	73	141	33	45	25	111	95	0.21	0.461
Skrabic et al. [59]	66	52	16	184	84	51	66	15	168	96	0.35	0.363
Turpeinen (Turku) et al. [36]	35	106	57	176	220	152	441	204	745	849	0.001	0.532
Turpeinen (Tampere) et al. [36]	13	23	20	49	63	69	229	152	367	533	0.25	0.592
Turpeinen (Oulu) et al. [36]	43	115	81	201	277	165	389	289	719	967	0.09	0.573
Bianco et al. [60]	18	11	2	47	15	11	20	5	42	30	0.39	0.416
San Pedro et al. [38]	15	37	19	67	75	28	43	17	99	77	0.94	0.437
Garcia et al. [61]	54	115	44	223	203	43	125	35	211	195	< 0.001	0.48
Lemos et al. [41]	55	100	50	210	200	68	101	63	237	227	0.04	0.489
Israni et al. [42]	85	133	18	303	169	60	110	27	230	164	0.03	0.416
Panierakis et al. [15]	37	57	6	131	69	23	58	15	104	88	0.03	0.458
Yavuz et al. [44]	36	58	23	130	104	35	70	31	140	132	0.72	0.485
Bonakdaran et al. [46]	13	52	4	78	60	18	26	1	62	28	0.01	0.311
Mohammadnejad et al. [48]	27	48	12	102	72	27	57	16	111	89	0.12	0.445
Greer et al. [50]	15	24	11	54	46	12	32	11	56	54	0.22	0.49
Abd-Allah et al. [52]	44	65	11	153	87	36	68	16	140	100	0.06	0.416
Cheon et al. [62]	5	32	44	42	120	9	34	70	52	174	0.1	0.769
Khalid et al. [63]	49	44	7	142	58	26	21	3	73	27	0.64	0.27
Nasreen et al. [54]	14	25	5	53	35	15	25	4	55	33	0.15	0.375
Iyer et al. [64]	17	16	17	50	50	18	16	16	52	48	0.01	0.48
Mukhtar et al. [55]	43	26	33	112	92	86	0	14	172	28	< 0.001	0.14
Rasoul et al. [57]	192	31	29	415	89	162	37	15	361	67	< 0.001	0.156
Ahmed et al. [65]	24	22	4	70	30	37	13	0	87	13	< 0.001	0.15

*P-HWE P* value for Hardy–Weinberg equilibrium, *MAF* minor allele frequency of control group

### Quantitative synthesis

#### Meta-analysis of the association between FokI (rs2228570) polymorphism and T1DM risk

**Fig. 2:**
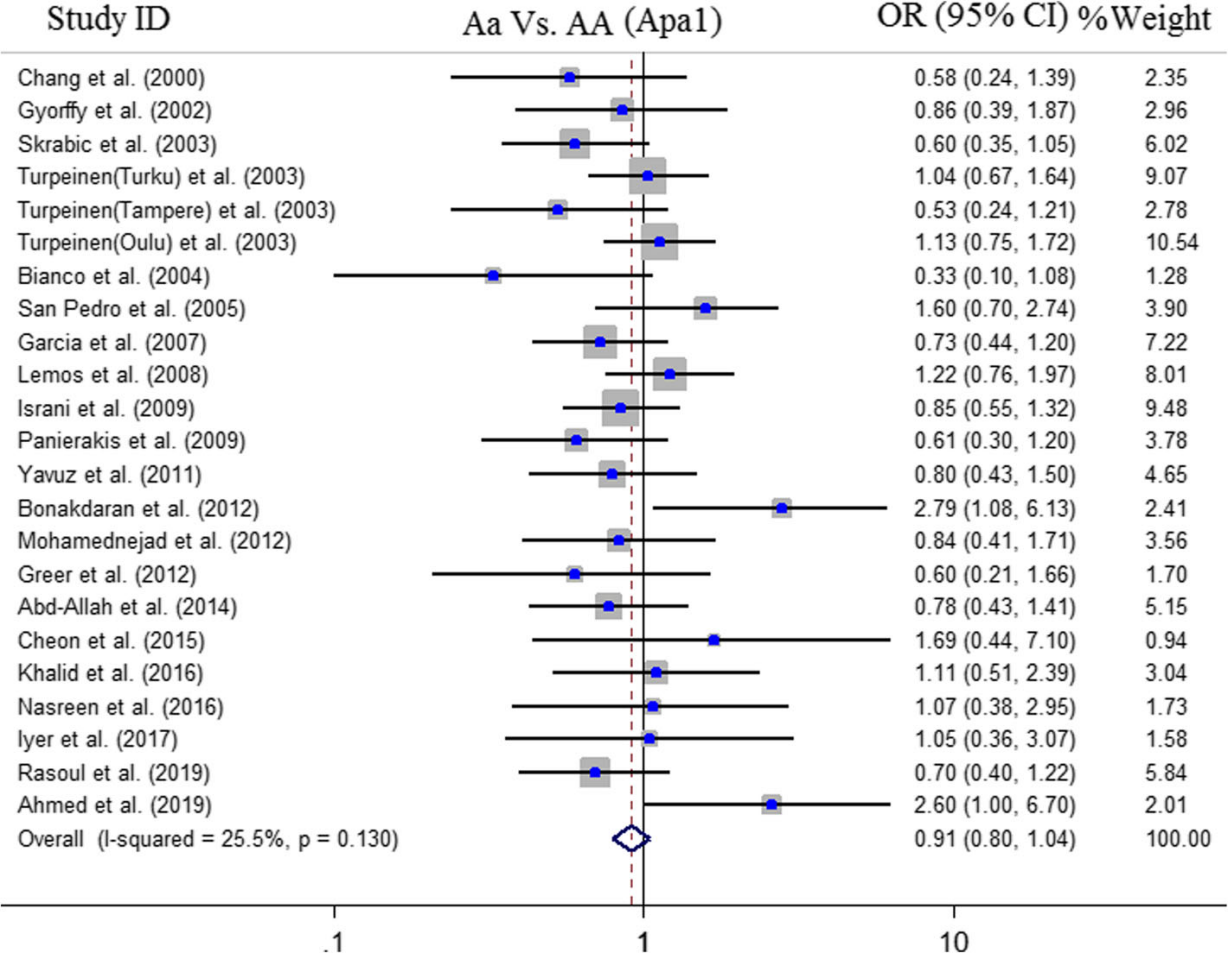
Pooled OR and 95% CI of individual studies and pooled data for the association between *ApaI* gene polymorphism and T1DM risk in heterozygote contrast (Aa vs. AA)

Overall, 29 case-control studies with 3723 cases and 5578 controls were analyzed for assessment of FokI polymorphism and T1DM risk. Of 29 studies, 15 studies were conducted in European countries [15, 34–36, 38–41, 44, 47, 49, 71], 9 studies were in Asian countries [33, 42, 45, 46, 48, 54–57], 3 studies were in African population [51–53] and eventually one study in Australia [50] and one study in American population [43]. Among studies were performed in Europe, Audi et al. [71] conducted an association study in different city of Spain (Barcelona and Navarra) and reported all data separately including genotype and allele frequency; thus we considered each population as a separate study. The pooled results revealed no significant association in overall population across all genotype models, meanwhile subgroup analysis according to ethnicity showed decreased risk of T1DM susceptibility in European population [dominant model (OR = 0.86, 95% CI, 0.74–1.00, *P* = 0.05) and heterozygote contrast (OR = 0.86, 95% CI, 0.75–0.99, *P* = 0.04)] and increased risk of T1DM susceptibility in African population under all genotype models; dominant model (OR = 2.06, 95% CI, 1.20–3.53, *P* = 0.008), recessive model (OR = 2.14, 95% CI, 1.03–4.43, *P* = 0.04), allelic model (OR = 1.17, 95% CI, 1.06–2.97, *P* = 0.02), ff vs. FF model (OR = 3.11, 95% CI, 1.44–6.69, *P* = 0.004), and Ff vs. FF model (OR = 1.81, 95% CI, 1.13–2.91, *P* = 0.01). Besides, susceptibility to T1DM in Asians compared to Africans and Europeans were not affected by FokI polymorphism (Fig. 2). The results of pooled ORs, heterogeneity tests and publication bias tests in different analysis models are shown in Table 3.

**Table 3:** Main results of pooled ORs in meta-analysis of Vitamin D Receptor gene polymorphisms

Group	Genetic Model	Case/Control	Test of Association		Test of Heterogenicity		Test of publication bias			
							(Begg’s test)		(Egger’s test)	
			OR	95%CI (***P*** value)	***I***^***2***^ (%)	***P***	Z	P	T	***P***
**FokI (rs2228570)**										
**Overall**	Dominant model	3723 / 5578	0.92	0.79–1.08 (0.31)	< 0.001	< 0.001	0.28	0.78	0.79	0.43
	Recessive model	3723 / 5578	0.98	0.71–1.35 (0.91)	< 0.001	< 0.001	1.43	0.15	1.28	0.21
	Allelic model	3723 / 5578	0.96	0.81–1.14 (0.65)	< 0.001	< 0.001	0.71	0.47	0.87	0.39
	ff vs. FF	3723 / 5578	0.96	0.69–1.35 (0.83)	< 0.001	< 0.001	1.70	0.09	1.78	0.08
	Ff vs. FF	3723 / 5578	0.94	0.79–1.12 (0.49)	< 0.001	< 0.001	1.19	0.23	1.23	0.22
**European**	Dominant model	3723 / 5578	**0.86**	**0.74–1.00 (0.05)**	0.268	0.268	−0.15	0.88	0.33	0.74
	Recessive model	2077 / 3849	1.00	0.77–1.30 (0.98)	0.011	0.011	0.60	0.54	1.15	0.27
	Allelic model	2077 / 3849	0.93	0.82–1.06 (0.28)	0.015	0.015	- 0.05	0.96	0.69	0.50
	ff vs. FF	2077 / 3849	0.90	0.67–1.20 (0.46)	0.046	0.046	0.27	0.78	1.01	0.33
	**Ff vs. FF**	**2077 / 3849**	**0.86**	**0.75–0.99 (0.04)**	0.435	0.435	0.74	0.45	0.59	0.56
**Asian**	Dominant model	2077 / 3849	0.76	0.55–1.05 (0.09)	0.015	0.015	- 0.74	0.45	−0.31	0.76
	Recessive model	1107 / 1272	0.93	0.23–3.68 (0.91)	< 0.001	< 0.001	1.65	0.09	3.26	0.02
	Allelic model	1107 / 1272	0.78	0.46–1.33 (0.36)	< 0.001	< 0.001	− 0.25	0.80	0.04	0.97
	ff vs. FF	1107 / 1272	0.87	0.25–3.01 (0.82)	< 0.001	< 0.001	1.95	0.05	3.01	0.03
	Ff vs. FF	1107 / 1272	0.84	0.53–1.34 (0.47)	< 0.001	< 0.001	0.49	0.62	0.50	0.63
**African**	Dominant model	1107 / 1272	**2.06**	**1.20–3.53 (0.008)**	0.225	0.225	- 0.52	0.60	−0.19	0.88
	Recessive model	312 /220	**2.14**	**1.03–4.43 (0.04)**	0.382	0.382	- 0.52	0.60	−0.60	0.65
	Allelic model	312 /220	**1.77**	**1.06–2.97 (0.02)**	0.057	0.057	0.52	0.60	0.23	0.85
	ff vs. FF	312 /220	**3.11**	**1.44–6.69 (0.004)**	0.493	0.493	- 1.57	0.11	−1.65	0.34
	Ff vs. FF	312 /220	**1.81**	**1.13–2.91 (0.01)**	0.337	0.337	- 0.52	0.60	0.03	0.98
**TaqI (rs731236)**										
**Overall**	Dominant model	1873 / 1895	1.06	0.78 – 1.45 (0.70)	78.3	< 0.001	− 0.45	0.65	−1.61	0.12
	Recessive model	1873 / 1895	0.91	0.66 – 1.26(0.58)	59.1	0.001	−1.93	0.05	−1.93	0.07
	Allelic model	1873/ 1895	1.02	0.81 – 1.29 (0.86)	81.9	< 0.001	−0.24	0.80	− 0.96	0.34
	tt vs. TT	1873 / 1895	0.90	0.58 – 1.39 (0.62)	72.9	< 0.001	−2.14	0.03	−2.65	0.01
	Tt vs.TT	1873 / 18995	1.12	0.84– 1.49 (0.45)	70.7	< 0.001	−0.39	0.69	−1.04	0.31
**European**	Dominant model	840 / 878	0.82	0.59–1.13 (0.23)	49.1	0.056	−1.48	0.13	−1.88	0.11
	Recessive model	840 / 878	0.78	0.50–1.21 (0.26)	55.1	0.029	−1.24	0.21	−0.95	0.38
	Allelic model	840 / 878	0.92	0.76–1.11 (0.36)	9.6	0.356	−1.73	0.08	−1.27	0.25
	tt vs. TT	840 / 878	0.75	0.44–1.27 (0.28)	61.1	0.012	−1.73	0.08	−1.68	0.14
	Tt vs.TT	840 / 878	0.87	0.64–1.20 (0.40)	39.8	0.114	− 0.99	0.32	−1.10	0.31
**Asian**	Dominant model	1033 / 1017	1.40	0.75 – 2.58 (0.28)	85.7	< 0.001	0	1	−1.08	0.31
	Recessive model	1033 / 1017	1.05	0.51 – 2.16 (0.88)	74.5	0.008	−2.44	0.01	−3.55	0.02
	Allelic model	1033 / 1017	1.27	0.75 – 2.14 (0.36)	88.7	< 0.001	0	1	−0.75	0.45
	tt vs. TT	1033 / 1017	1.03	0.37 – 2.85 (0.95)	85.4	< 0.001	−1.69	0.09	−3.10	0.03
	Tt vs.TT	1033 / 1017	1.46	0.83 – 2.58 (0.19)	80.1	< 0.001	− 0.83	0.40	− 0.77	0.46
**BsmI (rs1544410)**										
**Overall**	Dominant model	4826 / 7159	1.02	0.80– 1.30 (0.88)	76.3	< 0.001	−0.25	0.80	0.48	0.63
	Recessive model	4826 / 7159	0.94	0.80 – 1.10 (0.45)	52.9	< 0.001	0.13	0.89	0.20	0.84
	Allelic model	4826 / 7159	0.99	0.86 – 1.15 (0.92)	77.6	< 0.001	0.21	0.83	0.16	0.87
	bb vs. BB	4826 / 7159	0.96	0.75– 1.23 (0.74)	59.8	< 0.001	−0.59	−0.55	−0.69	0.49
	Bb vs. BB	4826 / 7159	1.07	0.88 – 1.29 (0.52)	53.9	< 0.001	−0.19	0.84	−0.58	0.56
**European**	Dominant model	1938 / 4450	0.94	0.71–1.24 (0.66)	71.0	< 0.001	−0.25	0.80	0.89	0.39
	Recessive model	1938 / 4450	1.00	0.85–1.19 (0.95)	20.7	0.223	−0.25	0.80	−0.63	0.54
	Allelic model	1938 / 4450	1.00	0.89–1.13 (0.93)	41.7	0.046	−0.35	0.72	−0.75	0.46
	bb vs. BB	1938 / 4450	0.99	0.80–1.23 (0.92)	16.1	0.273	0.05	0.96	−0.57	0.57
	Bb vs. BB	1938 / 4450	1.05	0.89–1.25 (0.56)	15.0	0.286	−0.45	0.65	−0.99	0.34
**Asian**	Dominant model	2195 /2004	1.05	0.61 – 1.79 (0.87)	77.8	< 0.001	− 0.12	0.90	−0.38	0.71
	Recessive model	2195 /2004	1.02	0.73 – 1.40 (0.92)	65.7	< 0.001	−0.38	0.70	0.18	0.86
	Allelic model	2195 /2004	1.00	0.72 – 1.38 (0.97)	85	< 0.001	0.38	0.70	0.24	0.81
	bb vs. BB	2195 /2004	1.07	0.55 – 2.09 (0.84)	76.8	< 0.001	−0.12	0.90	−0.42	0.68
	Bb vs. BB	2195 /2004	1.07	0.67 – 1.71(0.77)	63.5	< 0.001	0.12	0.90	−0.49	0.63
**American**	Dominant model	463 / 479	**0.57**	**0.39–0.84 (0.004)**	0.0	0.755	1.57	0.11	14.1	0.04
	Recessive model	463 / 479	**0.62**	**0.41–0.94 (0.02)**	50.5	0.133	0.52	0.60	0.38	0.76
	Allelic model	463 / 479	**0.66**	**0.54–0.81 (< 0.001)**	0.0	0.549	0.52	0.60	0.80	0.57
	bb vs. BB	463 / 479	**0.52**	**0.34–0.80 (0.003)**	0.0	0.876	0.52	0.60	0.06	0.96
	Bb vs. BB	463 / 479	0.66	0.41–1.05 (0.08)	13.2	0.316	0.52	0.60	1.56	0.36
**African**	Dominant model	230 / 226	2.41	0.63–9.18 (0.19)	81	0.065	−0.52	0.60	−0.15	0.90
	Recessive model	230 / 226	0.99	0.52–1.89 (0.96)	26.8	0.242	−1	0.31	0.18	0.23
	Allelic model	230 / 226	1.63	0.65–4.08 (0.29)	86.3	0.031	−0.52	0.60	0.05	0.96
	bb vs. BB	230 / 226	1.18	0.26–5.25 (0.83)	67.0	0.082	−1	0.31	0.15	0.35
	Bb vs. BB	230 / 226	2.40	0.81–7.17 (0.11)	63.9	0.141	−0.52	0.60	−0.16	0.89
**ApaI (rs7975232)**										
**Overall**	Dominant model	2436 / 4074	1.03	0.82–1.29 (0.79)	66.2	< 0.001	0.25	0.80	0.62	0.54
	Recessive model	2436 / 4074	1.03	0.90–1.17 (0.68)	48.4	0.005	0.24	0.81	0.20	0.84
	Allelic model	2436 / 4074	1.05	0.90–1.23 (0.52)	72.7	< 0.001	0.99	0.32	0.98	0.34
	aa vs. AA	2436 / 4074	1.02	0.77–1.33 (0.90)	52.9	0.002	−0.18	0.85	−0.56	0.57
	Aa vs. AA	2436 / 4074	0.91	0.80–1.04 (0.18)	25.5	0.355	−0.03	0.97	0.05	0.97
**European**	Dominant model	1258/ 2913	0.91	0.70–1.18 (0.47)	49.1	0.039	−0.98	0.32	−1.24	0.25
	Recessive model	1258/ 2913	1.09	0.92–1.30 (0.32)	56.9	0.013	−0.63	0.53	−0.28	0.78
	Allelic model	1258/ 2913	0.99	0.81–1.21 (0.90)	68.6	0.001	−1.16	0.24	−0.62	0.54
	aa vs. AA	1258/ 2913	1.02	0.72–1.45 (0.91)	53.1	0.024	−1.70	0.08	−1.03	0.33
	Aa vs. AA	1258/ 2913	0.90	0.75–1.09 (0.29)	29.5	0.174	−1.70	0.08	−2.23	0.05
**Asian**	Dominant model	1178 / 1161	1.27	0.78–2.05 (0.34)	77.4	< 0.001	1.70	0.08	0.90	0.39
	Recessive model	1178 / 1161	0.91	0.71–1.15 (0.42)	52.0	0.027	1.88	0.06	1.26	0.24
	Allelic model	1178 / 1161	1.15	0.82–1.62 (0.40)	82.2	< 0.001	1.34	0.18	1.69	0.13
	aa vs. AA	1178 / 1161	1.14	0.63–2.04 (0.66)	64.8	0.002	1.34	0.18	0.23	0.82
	Aa vs. AA	1178 / 1161	0.92	0.72–1.18 (0.52)	6.8	0.379	1.46	0.14	1.35	0.22

#### Meta-analysis of the association between TaqI (rs731236) polymorphism and T1DM risk

There were 20 case-control studies with 1837 cases and 1895 controls concerning TaqI polymorphism and T1DM risk. Studies were performed in different population, 8 studies were in Europeans [15, 34, 35, 38, 41, 44, 59, 60], 8 studies in Asians [42, 46, 48, 57, 58, 62–64], 2 studies in Africans [52, 65] and one study each was in Australia [50] and Americans [61]. Meta-analysis rejected any significant association between TaqI SNP and the risk of T1DM susceptibility. Moreover, the results of subgroup analysis by ethnicity were not significant under five genotype models. In subgroup analysis, since there was only one study for the Australians [50], Americans [61], and two studies for Africans [52, 65], these studies were excluded from the analysis. The results of pooled ORs, heterogeneity tests and publication bias tests in different analysis models are shown in Table 3.

#### Meta-analysis of the association between BsmI (rs1544410) polymorphism and T1DM risk

**Fig. 3:**
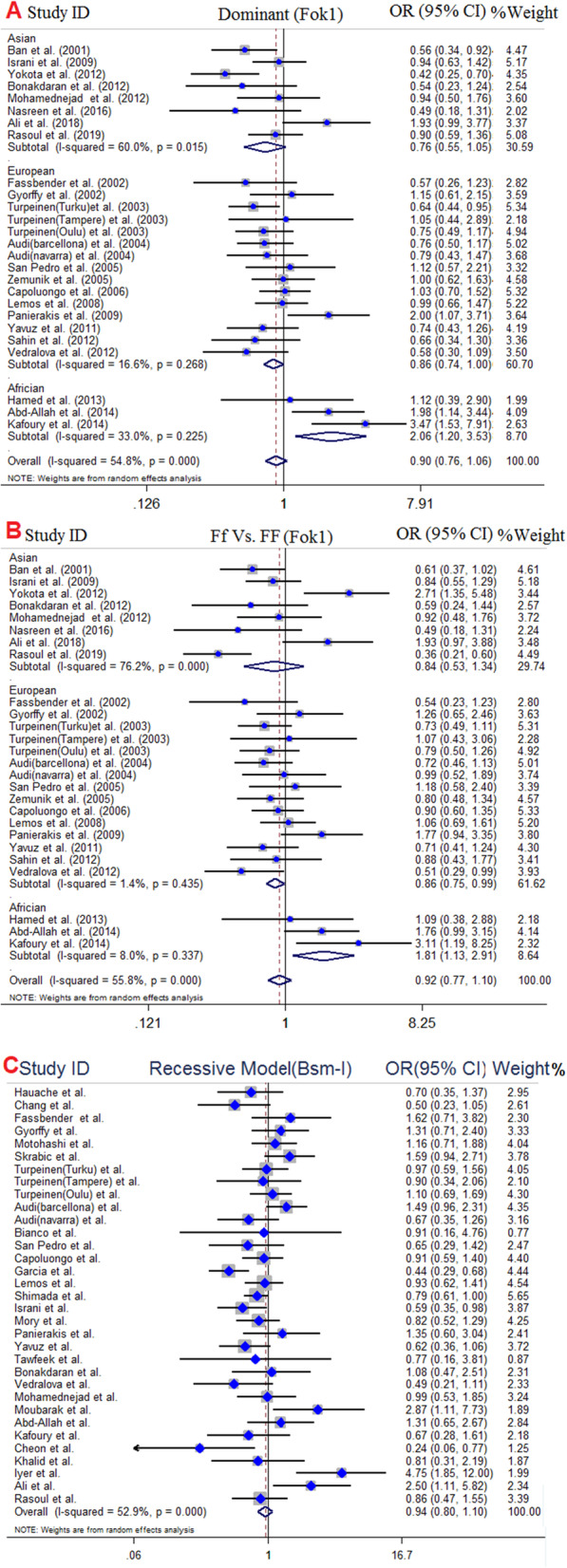
Pooled odds ratio (OR) and 95% confidence interval of individual studies and pooled data for the association between FokI, BsmI gene polymorphism and T1DM risk in different ethnicity subgroups and overall populations for A; dominant model (FokI), B; Ff vs. FF Model (FokI), and C; Recessive Model (BsmI)

To examining the association between *BsmI* polymorphism and T1DM risk, 34 case-control studies with 4826 cases and 7159 controls subjects were included. It was detected that 15 studies with 1938 cases and 4450 controls were performed in European countries [15, 34–36, 38, 40, 41, 44, 49, 59, 60, 71] which among these 15 studies, Turpeinen et al. [36] conducted an association study in different city of Finland (Turku, Tampere and Oulu) and reported all data separately, including genotype and allele frequency; thus we considered each population as a separate study. Moreover, 13 studies out of 34 eligible studies were carried out in Asian populations [42, 46, 48, 56–58, 62–64, 67–70], 3 studies were in Americans [43, 61, 66] and three studies were in Africans [52, 53, 65]. No significant association between BsmI polymorphism and T1DM risk were found under all genotype models for the overall population. However, pooled results of subgroup analysis indicated markedly significant negative associations between BsmI SNP and the risk of T1DM susceptibility in American populations across all genotype models; dominant model (OR = 0.57, 95% CI, 0.39–0.84, *P* = 0.004), recessive model (OR = 0.62, 95% CI, 0.41–0.94, *P* = 0.02), allelic model (OR = 0.66, 95% CI, 0.54–0.81, *P* < 0.001), bb vs. BB model (OR = 0.52, 95% CI, 0.34–0.80, *P* = 0.003), except Bb vs. BB model (OR = 0.66, 95% CI, 0.41–1.05, *P* = 0.08) (Fig. 3). No significant association was detected for European, Asian and African population. The results of pooled ORs, heterogeneity tests and publication bias tests in different analysis models are shown in Table 3.

#### Meta-analysis of the association between ApaI (rs7975232) polymorphism and T1DM risk

Finally, 24 case-control studies with 2436 cases and 4074 controls were identified eligible for quantitative synthesis of the association between ApaI polymorphism and T1DM risk. Overall, 10 studies were conducted in Europe [15, 35, 36, 38, 41, 44, 59, 60], 10 studies were in Asia [42, 46, 48, 54, 55, 57, 58, 62–64], 2 studies in Africa [52, 65] and one study each was in Australia [50] and America [61]. Because of limited number of studies performed in Australia, America and Africa these studies were excluded from subgroup analysis. The results demonstrated no significant association between the ApaI polymorphism and risk of T1DM in the overall population and ethnic-specific analysis (Fig. 3). The results of pooled ORs, heterogeneity tests and publication bias tests in different analysis models are shown in Table 3.

### Evaluation of heterogeneity and publication bias

**Fig. 4:**
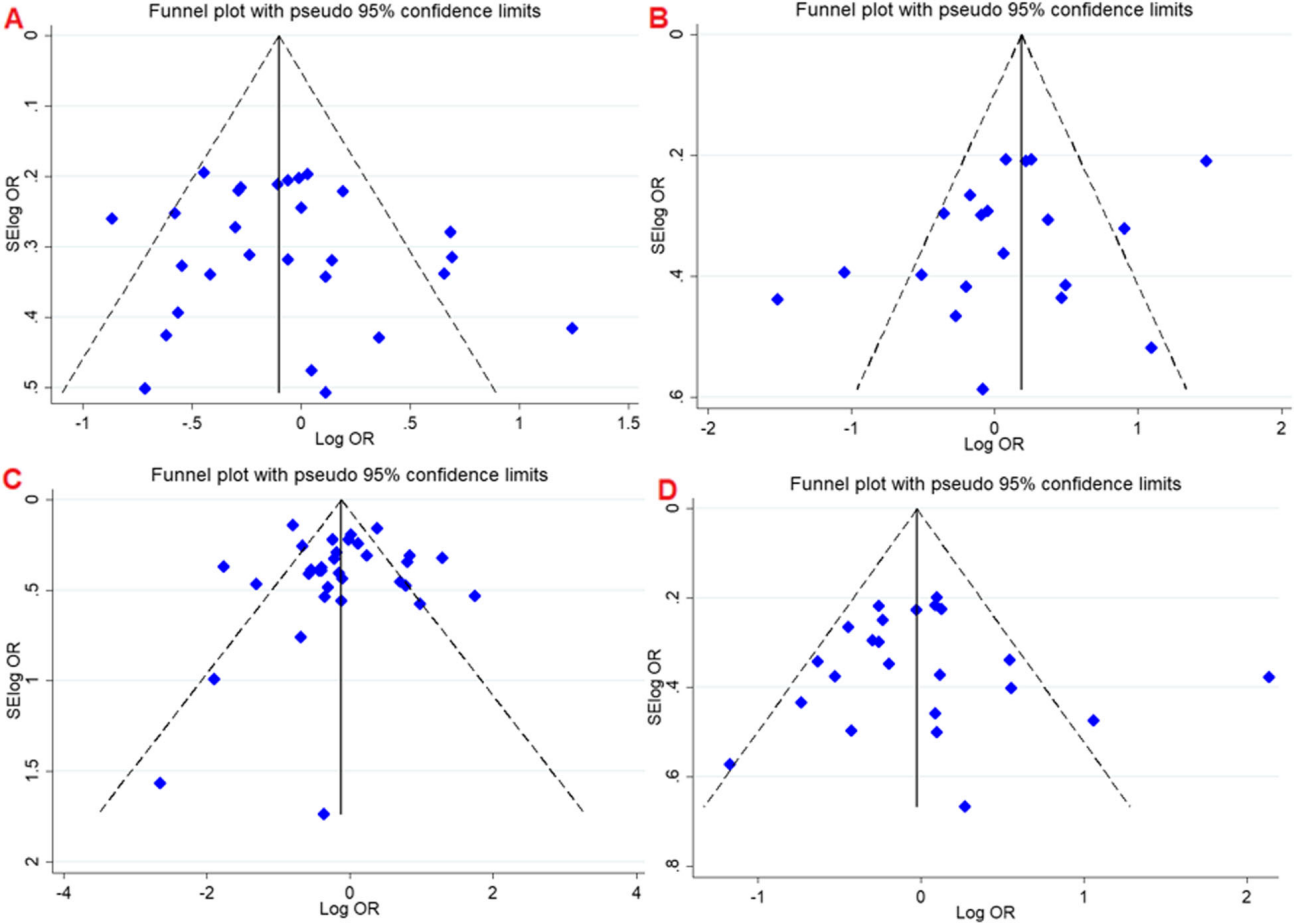
Begg’s funnel plot for publication bias test. A; dominant model *FokI*. B; dominant model *TaqI*. C; dominant model *BsmI.* D; dominant model *ApaI*. Each point represents a separate study for the indicated association

During the meta-analysis of *VDR* gene polymorphism evidence of substantial to moderate heterogeneity was detected. However, partial heterogeneity was resolved while the data were stratified by ethnicity. Publication bias was evaluated by funnel plot, Begg’s test and Egger’s test. There was no obvious evidence of asymmetry from the shapes of the funnel plots (Fig. 4), and all *P* values of Begg’s test and Egger’s test were > 0.05, which showed no evidences of publication biases.

### Sensitivity analysis

**Fig. 5:**
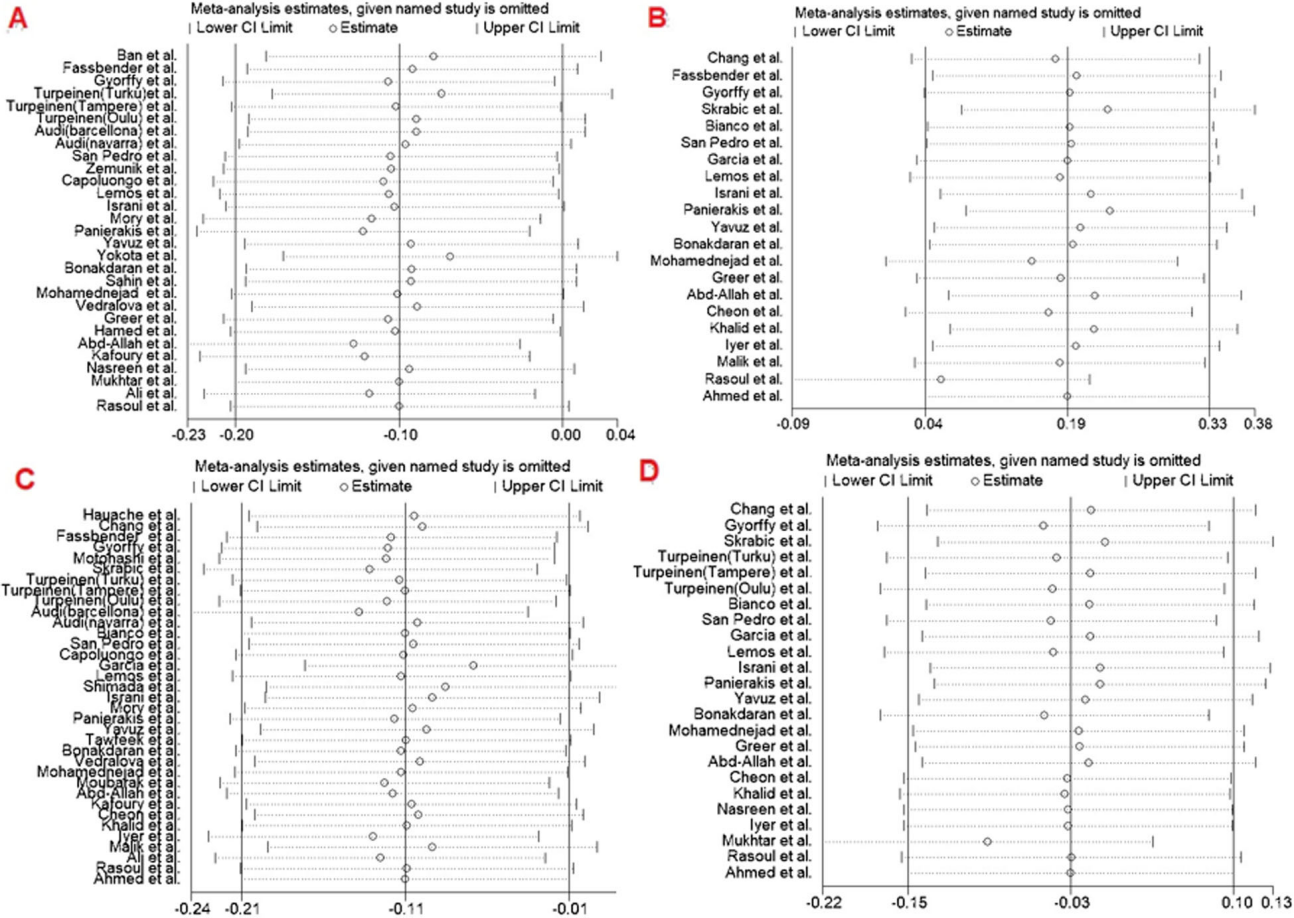
Sensitivity analysis in present meta-analysis investigates the single nucleotide polymorphisms of Vitamin D Receptor contribute to risk for T1DM (A, *FokI*; B, *TaqI*; C, *BsmI*; D*, ApaI*)

The leave-one-out method was used in the sensitivity analysis to explore the effect of individual data on the pooled ORs. The significance of ORs was not altered through omitting any single study in the dominant model for FokI, TaqI, BsmI and ApaI SNPs, indicating that our results were statistically robust (Fig. 5).

### Bayesian meta-regression analysis

**Fig. 6:**
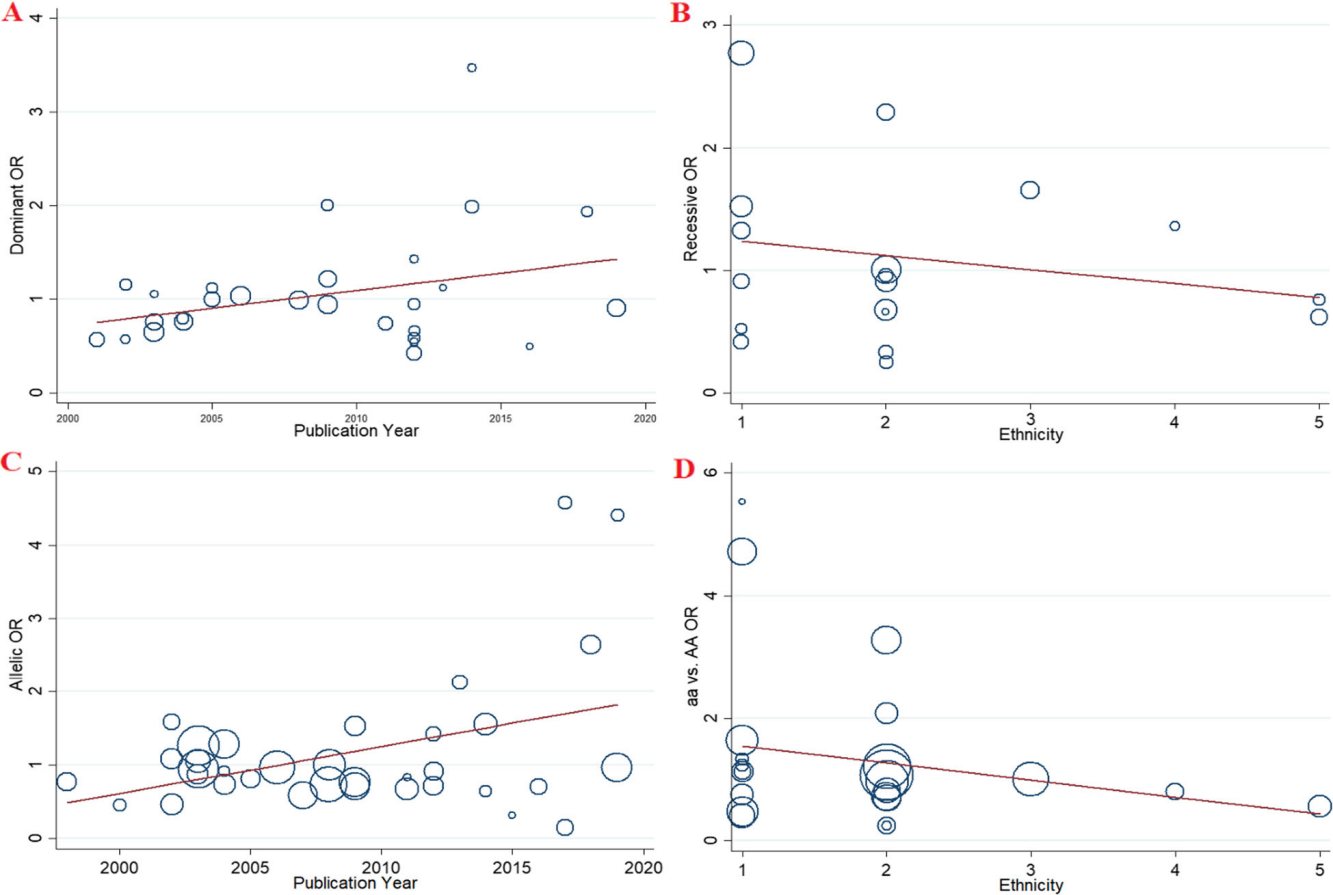
Meta-regression plots of the association between *VDR* gene polymorphisms and risk of CAD based on; A: Publication year (Dominant model), B: Ethnicity (Recessive model), C: Publication year (Allelic model), C: Ethnicity (aa vs. AA model)

Meta-regression and subgroup analyses were performed to explore potential sources of heterogeneity among included studies (Table 4). The findings of meta-regression indicated that ethnicity can be the potential source of heterogeneity, therefore, subgroup analysis was performed to attenuate the effect of these parameters. (Fig. 6).

**Table 4:** Meta-regression analyses of potential source of heterogeneity

Heterogeneity Factor		Coefficient	SE	T	***P***-value	95% CI	
						UL	LL
**FokI (rs2228570)**							
**Publication Year**	Dominant model	0.037	0.021	1.74	0.09	- 0.006	0.082
	Recessive model	0.763	0.313	2.44	0.02	0.117	1.410
	Allelic model	0.037	0.018	2.07	0.04	0.001	0.074
	ff vs. FF	0.631	0.242	2.60	0.01	0.130	1.131
	Ff vs. FF	0.032	0.022	1.43	0.16	−0.014	0.078
**Ethnicity**	Dominant model	0.322	0.081	3.97	0.001	0.155	0.489
	Recessive model	−1.10	1.43	−0.77	0.44	−4.063	1.85
	Allelic model	0.231	0.073	3.15	0.004	0.080	0.382
	ff VS. FF	−0.591	1.134	−0.52	0.60	−2.932	1.749
	Ff vs. FF	0.217	0.097	2.23	0.03	0.017	0.416
**TaqI (rs731236)**							
**Publication Year**	Dominant model	0.069	0.037	1.83	0.08	−0.010	0.148
	Recessive model	0.020	0.031	0.65	0.52	−0.046	0.087
	Allelic model	0.038	0.026	1.47	0.15	−0.016	0.093
	tt vs. TT	0.063	0.048	1.32	0.20	−0.039	0.166
	Tt vs.TT	0.064	0.037	1.72	0.10	−0.014	0.142
**Ethnicity**	Dominant model	−0.249	0.207	−1.20	0.24	−0.684	0.185
	Recessive model	−0.114	0.145	−0.79	0.44	− 0.424	0.194
	Allelic model	−0.145	0.123	−1.18	0.25	−0.404	0.113
	tt vs. TT	−0.167	0.253	−0.66	0.51	−0.707	0.373
	Tt vs.TT	−0.250	0.200	−1.25	0.22	−0.670	0.170
**BsmI (rs1544410)**							
**Publication Year**	Dominant model	0.142	0.046	3.03	0.005	0.046	0.237
	Recessive model	0.031	0.024	1.29	0.20	−0.018	0.081
	Allelic model	0.063	0.025	2.54	0.01	0.012	0.115
	bb vs. BB	0.103	0.047	2.17	0.03	0.006	0.200
	Bb vs. BB	0.095	0.033	2.84	0.008	0.026	0.163
**Ethnicity**	Dominant model	0.482	0.265	1.82	0.07	−0.058	1.023
	Recessive model	−0.133	0.139	−0.96	0.34	−0.417	0.149
	Allelic model	0.152	0.143	1.07	0.293	−0.138	0.444
	bb vs. BB	−0.274	0.280	−0.98	0.33	−0.846	0.296
	Bb vs. BB	0.381	0.188	2.03	0.05	−0.002	0.764
**ApaI (rs7975232)**							
**Publication Year**	Dominant model	0.098	0.054	1.81	0.08	−0.014	0.211
	Recessive model	0.005	0.030	0.18	0.86	−0.057	0.068
	Allelic model	0.052	0.032	1.64	0.11	−0.013	0.119
	aa vs. AA	0.042	0.042	0.98	0.33	−0.047	0.131
	Aa vs. AA	0.027	0.019	1.37	0.18	−0.014	0.069
**Ethnicity**	Dominant model	−0.130	0.290	−0.45	0.65	−0.733	0.471
	Recessive model	−0.086	0.175	−0.49	0.62	−0.452	0.279
	Allelic model	0.007	0.171	0.04	0.96	−0.348	0.362
	aa vs. AA	−0.279	0.243	−1.15	0.26	−0.785	0.226
	Aa vs. AA	0.033	0.103	0.32	0.74	−0.181	0.248

## Discussion

In this study, we performed a systematic review and meta-analysis to achieve a vivid and exact approximation of the associations between the *VDR* gene polymorphisms, including FokI (rs2228570), TaqI (rs731236), BsmI (rs1544410), and ApaI (rs7975232) and susceptibility to T1DM. The findings of meta-analysis on 39 case–control studies, containing 29 studies with 3723 cases and 5578 controls for FokI, 20 studies with 1837 cases and 1895 controls for TaqI, 34 studies with 4826 cases and 7159 controls for BsmI, and 24 studies with 2436 cases and 4074 controls for ApaI, indicated no significant association of *VDR* gene polymorphisms with T1DM risk in overall population. That notwithstanding, the subgroup analysis resulted in identification of significant associations between FokI and BsmI polymorphism and T1DM in African and American population. Our study provided some beneficial points over previous studies. First, this meta-analysis included further studies with more sample size compared with the previous studies, conferring more conclusive results. Second, we performed subgroup analysis by ethnicity to indicated association of *VDR* gene polymorphisms with T1DM risk in different ethnical groups.

Over the course of past years, a bulk of studies has addressed the association of *VDR* gene polymorphisms and risk of T1DM throughout various populations, resulting in conflicting findings [61, 67]. Such discrepancies might stem from diversity in detection methods, differences in diagnostic criterions, clinical heterogeneity, small sample sizes, low statistical power, and interactions between genetic and environmental contributing factors according to variations in the geo-epidemiological factors. As a consequence, three previous meta-analyses by Guo et al. [21] in 2006 [including 11 studies for FokI (1424 cases and 3301 controls), 13 studies for BsmI (1601cases and 4207 controls), 9 studies for ApaI (1101 cases and 2805 controls), and 7 studies for TaqI (681 cases and 781 controls)], Zhang et al. [22] in 2012 [T1DM cases and 4049 controls in 21 studies for BsmI, 2167 T1DM cases and 3402 controls in 17 studies for FokI, 1166 T1DM cases and 2328 controls in 11 studies for ApaI, and 1041 T1DM cases and 1137 controls in 8 studies for TaqI], and Tizaouia et al. [20] in 2014 (13 studies for TaqI, 23 studies for BsmI, 15 studies for ApaI, and 18 studies for FokI) were carried out to resolved the conundrum and attain an exact approximation. They indicated that *VDR* gene SNPs were not associated with T1DM risk, except than BsmI polymorphism association with T1DM predisposition that was observed in Zhang et al. [22] study. Upon the latest meta-analysis published in 2014, several original association studies evaluated the role of *VDR* gene polymorphisms with T1DM risk. As a result, the necessity for performing an updated meta-analysis is sensed to come up with resolution of the limitations of individual association studies and to gain a much more valid and comprehensive pooled estimation on the association of *VDR* gene polymorphisms with T1D risk.

Previous meta-analysis performed by Tizaouia et al. [20] in 2014 reported no significant association of *VDR* gene FokI polymorphism with risk of T1D. According to our meta-analysis, the pooled results in overall population across all genotype models demonstrated no significant association of *VDR* gene FokI polymorphism; nonetheless, subgroup analysis according to ethnicity showed a marginally-significant decreased susceptibility to T1DM in European population according to dominant genetic model and heterozygote comparison, while an increased risk of T1DM in African population according to all genotype models. In addition, our meta-analysis did not support any significant association between TaqI SNP and susceptibility to T1DM. Furthermore, the results of subgroup analysis according to ethnicity did not show any significant association in all genetic models. However, in the subgroup analysis, given that there was only one study in the Australian [50] and American [61] populations, and two studies in the African [52, 65] population, the subgroup analysis was not performed in these populations. In line with our findings, previous meta-analysis by Tizaouia et al. [20] also did not show significant association of *VDR* gene TaqI polymorphism with risk of T1D. According to the previous meta-analysis, BsmI SNP was not the risk factor for T1D susceptibility. However, after excluding one study, a marginal significant (*P* = 0.051) association was found in the homozygous model. On the other side, our meta-analysis also revealed that BsmI polymorphism was not a risk for T1DM in all genetic models when all of the population were analyzed. Nonetheless, subgroup analysis demonstrated a strong negative significant association between BsmI SNP and the risk of T1DM in American population in all of the genetic model comparisons. Finally neither our meta-analysis nor the previous one by Tizaouia et al. [20] found any significant association of ApaI polymorphism and T1DM risk in overall as well as subgroup analyses. Taken together, although our meta-analysis included further studies compared to the previous study, the overall analysis was almost the same. Nonetheless, our subgroup analysis indicated association of *VDR* genetic polymorphisms with T1DM risk in different ethnical groups.

In their meta-analysis, Tizaoui et al. [20] indicated in the stratification analysis that publication year, age, gender, estimated VitD levels, and latitude modulated the association between *VDR* gene polymorphisms and T1D risk. Furthermore, another meta-analysis revealed a relationship between winter ultraviolet radiation (UVR) and *VDR* gene polymorphisms in T1DM, implying to the influence of the UVR on the association between *VDR* polymorphisms and T1DM susceptibility [72]. During the four cooler months, it was observed that latitude strongly determines the available levels of VitD producing UV. As latitude increases, the amount of VitD producing UV decreases, which may prevent VitD synthesis in humans [73]. As a result, the latitude of the locations in which the individuals live may impress the susceptibility to develop T1DM.

Despite we tried to conduct best meta-analysis of the VDR gene polymorphisms and susceptibility to RA, there was also a number of limitations that should be taken into account. First, there was significant heterogeneity across studies, which may lessen the certainty of the results. However, we tried to find and attenuate its effect by meta-regression and subgroup analysis. Consequently, heterogeneity was still an unavoidable problem that may influence the accuracy of the overall results. Second, only articles published in the English language were include in this meta-analysis. Third, our meta-analysis was based on crude approximation of the genetic variations regardless of adjusting the analysis by gender, age, VitD intake, and other environmental factors like exposure to sun light, as several studies noted the involvement of these parameters as well as gene-environment and gene-gene interactions in the susceptibility and of RA and we could not analyze it owing to a lack of published well-structured data.

## Conclusion

In conclusion, this study was a systematic review and meta-analysis of 40 case–control association studies to come up with the clear estimation of the associations between the *VDR* gene SNPs [FokI (rs2228570), TaqI (rs731236), BsmI (rs1544410), and ApaI (rs7975232)] and susceptibility to T1DM. The findings of meta-analysis revealed no significant association of *VDR* gene SNPs with T1DM risk in the overall population. However, the subgroup analysis indicated significant associations between FokI and BsmI polymorphism and T1DM risk in African and American population. As a limitation, we did not evaluate a number of *VDR* gene SNPs that might act in interaction with environmental factors to determine the fate of T1DM pathogenicity. Further investigations on the VDR, above and beyond the genetic as well as traditional risk factors, may confer a possibility for identification of critical susceptibility factors in the disease development, which might be applicable in the personalized medicine for better and optimized therapy of T1DM patients.

## Data Availability

All data generated or analyzed during this study are included in this published article.

## Abbreviations

T1DM: Type 1 diabetes mellitus
VDR: Vitamin D receptor
Vitamin D: VitD
SNP: Single nucleotide polymorphisms
IL: Interleukin
PRISMA: Preferred Reporting Items for Systematic reviews and Meta-Analyses
NOS: Newcastle-Ottawa Scale
UVR: Ultraviolet radiation
Th: T helper
TNF: Tumor necrosis factor
IFN: Interferon
HWE: Hardy–Weinberg equilibrium
PCR- RFLP: Polymerase chain reaction-restriction fragment length polymorphism
